# Supplementary material for: Inversion symmetry and bulk Rashba effect in methylammonium lead iodide perovskite single crystals
Source: Nat Commun. 2018 May 8;9:1829. doi: 10.1038/s41467-018-04212-w (PMC5940805; doi:10.1038/s41467-018-04212-w)
Supplement: Supplementary file 1 — Supplementary Information [file 41467_2018_4212_MOESM1_ESM.pdf]

Supplementary Information for:
Inversion Symmetry and Bulk Rashba Effect in Methylammonium Lead Iodide Perovskite Single Crystals

Kyle Frohna et al.

Supplementary Note 1: Sample Details

The data presented in Fig. 1 were measured from the polished (100) surface of the $MAPbI_3$ single crystal as shown in the photograph in Supplementary Fig. 3 below. We measured five single crystals from the same growth batch, two with polished surfaces and three with as-grown surfaces. Only the two polished crystals yielded measurable SHG-RA patterns, which can most likely be attributed to the visibly better surface quality of the polished crystals. We also found that the SHG-RA patterns are not reproducible after thermal cycling across the cubic-to-tetragonal structural phase transition at 327 K, which may be due to crystalline imperfections enhanced at the surface. Such extreme sensitivity to surface quality further supports our interpretation that the observed SHG signal originates from a surface electric-dipole process.

Supplementary Figures

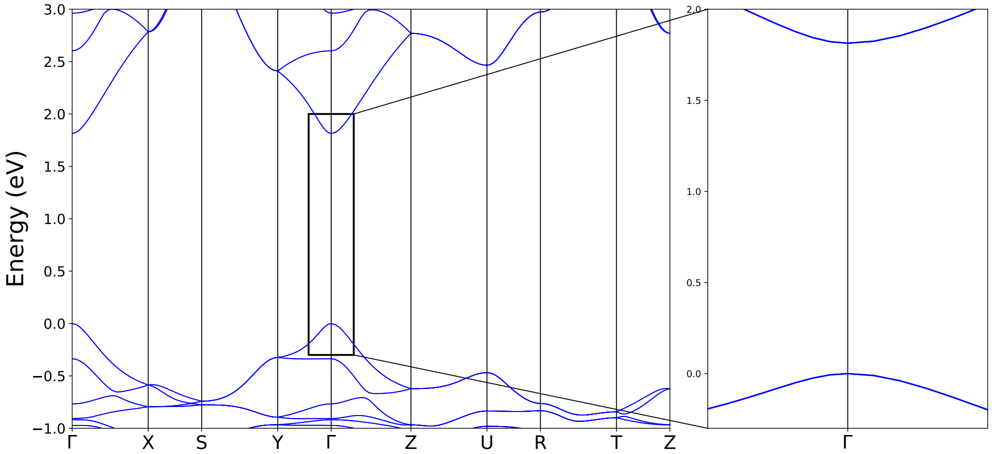

Supplementary Figure 1: **Fully relaxed orthorhombic $MAPbI_3$ bandstructure** Calculated bandstructure of an experimentally measured orthohombic unit $MAPbI_3$ structure[1] fully relaxed in DFT. No discernable Rashba splitting is observed in the band extrema

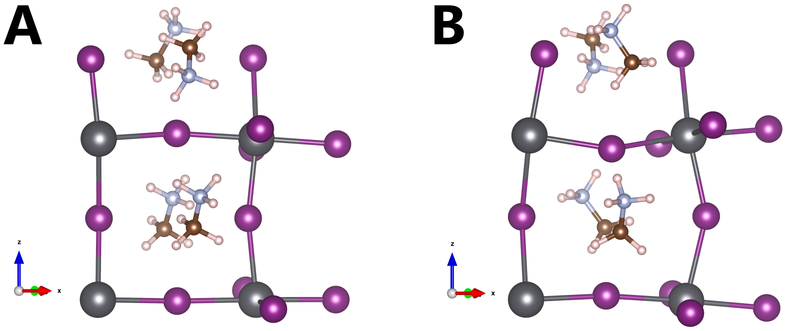

Supplementary Figure 2: **Additional DFT relaxed structures** The structures of initially cubic (**a**) and tetragonal (**b**) supercells after full DFT relaxation of the atomic positions with fixed lattice parameters.

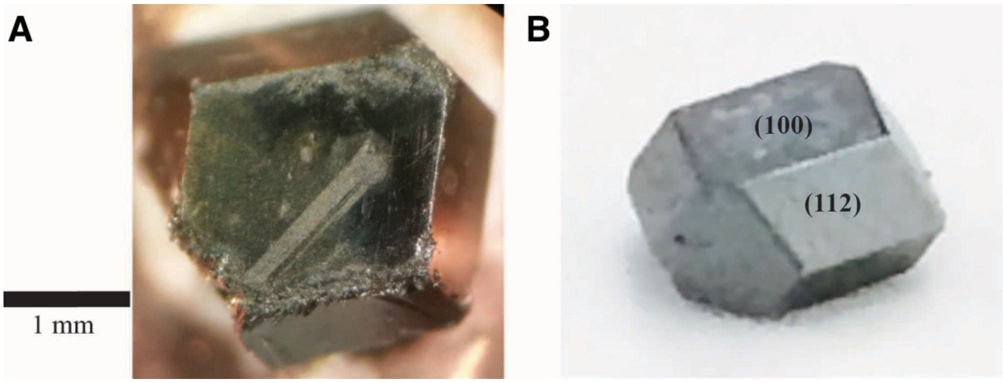

Supplementary Figure 3: **$MAPbI_3$ single crystal** Photograph of the $MAPbI_3$ single crystal on which the data in Fig. 1 was collected. Both (**A**) a zoomed-in view of the polished (100) surface used for SHG-RA measurements and (**B**) a zoomed-out view of the overall crystal morphology and surface orientations are shown.

Supplementary References

1. Whitfield, P. S. *et al.* Structures, phase transitions and tricritical behavior of the hybrid perovskite methyl ammonium lead iodide. *Scientific Reports* **6**, 35685 EP – (2016).
