# Supplementary material for: Inversion symmetry and bulk Rashba effect in methylammonium lead iodide perovskite single crystals
Source: Nat Commun. 2018 May 8;9:1829. doi: 10.1038/s41467-018-04212-w (PMC5940805; doi:10.1038/s41467-018-04212-w)
Supplement: Supplementary file 3 — Description of Additional Supplementary Info [file 41467_2018_4212_MOESM3_ESM.pdf]

Description of Additional Supplementary Files

File Name: Supplementary Dataset 1
Description: Partially relaxed cubic MAPbI3 structure. .cif file of a cubic MAPbI3 structure relaxed using the partial strategy described in the main text. The experimental structure and lattice constants are first taken and relaxed in DFT without constraints until the forces are reduced as far as possible without breaking the experimentally observed symmetry. Then, the lattice constants are set to these 'low pressure' values, the position of the lead-iodide framework is fixed and the MA cations are allowed to fully relax.

File Name: Supplementary Dataset 2
Description: Partially relaxed tetragonal MAPbI3 structure. .cif file of a tetragonal MAPbI3 structure relaxed using the partial strategy described in the main text. The experimental structure and lattice constants are first taken and relaxed in DFT without constraints until the forces are reduced as far as possible without breaking the experimentally observed symmetry. Then, the lattice constants are set to these 'low pressure' values, the position of the lead-iodide framework is fixed and the MA cations are allowed to fully relax.

File Name: Supplementary Dataset 3
Description: Fully relaxed orthorhombic MAPbI3 structure. .cif file of an orthorhombic MAPbI3 structure taken from experiment and subsequently fully relaxed in DFT without constraint.
